# The onset of hypertension in metabolic syndrome is independent of renal sympathetic innervation

**DOI:** 10.26508/lsa.202503568

**Published:** 2026-07-14

**Authors:** Omar Flores-Sandoval, Skarleth Cárdenas-Romero, Adrián Báez-Ruiz, Roberto C Salgado-Delgado, Nadia Saderi

**Affiliations:** 1 Laboratorio de Neuroanatomía Funcional, Facultad de Ciencias, Universidad Autónoma de San Luis Potosí , San Luis Potosí, México; 2 Laboratorio de Ritmos Biológicos, Facultad de Ciencias, Universidad Autónoma de San Luis Potosí , San Luis Potosí, México

## Abstract

Increased sympathetic tone and hypertension are hallmarks of metabolic syndrome and contribute to chronic kidney disease. Although renal sympathetic denervation transiently lowers blood pressure, its role in the development of metabolic and renal alterations remains unclear. Here, we evaluated the contribution of renal sympathetic input to the onset and progression of high-fat diet–induced alterations. Male Wistar rats underwent bilateral renal denervation before metabolic challenge and were fed a standard or high-fat diet for 8 or 12 wk. High-fat feeding induced hypertension, proteinuria, increased angiotensin II, and reduced creatinine clearance, urinary flow, and potassium excretion, independently of denervation. Renal norepinephrine content confirmed effective denervation and was not affected by diet. Denervation attenuated ketonuria in high-fat diet–fed rats. The phosphorylation of AKT, PI3K, and ERK1/2 in the kidney was modulated by interactions among diet, renal sympathetic input, and time. These findings indicate that renal sympathetic nerves contribute to early stages of metabolic dysregulation, whereas prolonged hypercaloric exposure overrides autonomic control and promotes cardiovascular and renal complications.

## Abstract (precis)

In a male rat model of metabolic syndrome, renal denervation before the high-fat diet has only a transient effect, suggesting that hypercaloric diet–induced endocrine changes trigger hypertension independently of renal sympathetic activity.

## Introduction

In 2021, the World Health Organization reported that 1.28 billion people in the world suffer from systemic arterial hypertension (HT) or high blood pressure (HBP) (Overweight & Obesity, CDC, 2024). Although nowadays the clinical practice avails itself of several antihypertensive agents, the prevalence of HT exceeds 50%, and a significant gap exists in diagnosis and therapy between high- and low-income countries. Regardless of the underlying etiology, the progression of HT results in organ failure, principally the kidneys and heart, leading to premature death (Al Ghorani et al, 2021). A large body of research links HT pathophysiology to the obesity epidemic because there is a nearly linear relationship between increased body mass index and HBP, with higher body mass index shifting the distribution toward higher blood pressure levels (Hall et al, 2019). The co-occurrence of HT and obesity involves several interconnected disorders, collectively known as metabolic syndrome (MetS) (Lin et al, 2022). Both clinical data and experimental animal studies indicate that HT, visceral fat accumulation (abdominal obesity), dyslipidemia, glucose intolerance, and insulin resistance are characterized by increased renal sympathetic nerve activity (RSNA) and a higher risk of chronic kidney disease (CKD) (Amann & Benz, 2013; Oparil et al, 2018; DeLalio et al, 2020). This has led to the hypothesis that disrupting the brain–kidney communication might prevent HT and renal failure. Radiofrequency ablation of sympathetic nerves has been proposed and repeatedly tried as a treatment for resistant HT. The positive results have led to US Food and Drug Administration approval of their different technologies, with beneficial results on blood pressure and metabolic balance (Mahfoud et al, 2011; Witkowski et al, 2011; Kampmann et al, 2017; Lee, 2020). However, several issues have emerged from clinical studies, and the long-term safety and effectiveness of renal denervation for controlling HBP and its effects on insulin handling in diabetic patients remain uncertain (Verloop et al, 2015; Miroslawska et al. 2016, 2021; Lohmeier & Hall, 2019; Grassi, 2021). In addition, increased renal spillover of norepinephrine (NE) has been observed in obese, normotensive individuals (Grassi et al, 1995; Vaz et al, 1997), suggesting that obesity itself promotes RSNA and can impair renal physiology independently of HT. Several researchers have provided evidence that MetS-related HT depends on sustained brain stimulation by systemic and local angiotensin II (ANG-II) signaling, along with adiposity signals like insulin and leptin, all of which promote sympathoexcitation (Rahmouni et al, 2003; Hilzendeger et al, 2012; Campos et al, 2015). The renin–angiotensin system (RAS) is a physiological mechanism that raises blood pressure through thirst stimulation, vasoconstriction, Na^+^ and H_2_O reabsorption, and sympathetic activation (Oparil et al, 2003). RAS activity is heightened in obesity and insulin resistance (Kumari et al, 2019). For example, a positive feedback loop has been described involving the type 1 angiotensin II receptor (AT1-R) and dyslipidemia, where ANG-II, the active product of the RAS cascade, stimulates low-density lipoprotein uptake and oxidation. Conversely, oxidized LDL increases AT1-R expression (Nickenig et al, 1997). ANG-II triggers several changes that predispose to renal injury, such as constriction of efferent arterioles and Na^+^ retention, by increasing glomerular filtration, regulating tubular transport proteins, and up-regulating sympathetic tone, vasopressin, and aldosterone secretion (Johnson & Malvin, 1977; Barton et al, 1997; Neuhofer & Pittrow, 2006; Harrison-Bernard, 2009). Elevated systemic and local ANG-II levels are also involved in the complex interplay among oxidative stress, endothelial dysfunction, chronic inflammation, and insulin resistance. The up-regulation of ANG-II signaling, oxidative stress, and kidney endothelial dysfunction correlates directly with insulinemia (Sarafidis & Ruilope, 2006). Moreover, insulin promotes renal cell proliferation, extracellular matrix production, and the release of growth factors, leading to fibrotic changes seen in CKD (Abrass et al. 1994, 1995). The role of leptin in mediating HT and CKD within MetS warrants further investigation. Nevertheless, plasma leptin levels are associated with sympathetic activity, including in the kidneys, and may enhance renal Na^+^ retention (Haynes et al, 1997; Shek et al, 1998; Carlyle et al, 2002; Kim et al, 2013). Interestingly, obese individuals with genetic leptin deficiency and sympathetic dysfunction also show a reduced RAS response to upright posture (Ozata et al, 1999). Given this background, the present study aims to explore how RSNA influences the early development of certain CKD parameters in a male animal model of MetS induced by a high-fat diet (HFD). To address this question, bilateral renal denervation (BRDx) was performed before exposure to the hypercaloric diet, allowing us to distinguish its role in the onset rather than the reversal of metabolic and renal dysfunction. We then compared blood pressure, metabolic and renal function parameters, and hormone signaling proteins (protein kinase B (Akt), phosphoinositide 3-kinase (PI3K), and extracellular signal-regulated kinase 1/2 (ERK1/2)) in male rats fed either a regular diet or an HFD for 8 and 12 wk.

## Results

### Effects of BRDx and HFD on body weight, and food and water intake

**Figure 1.**
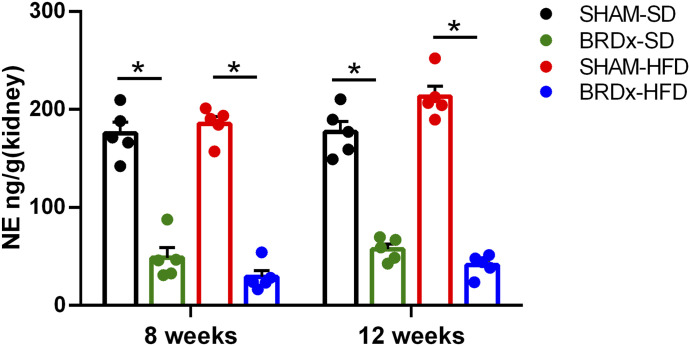
BRDx decreases the kidney content of NE. Quantification of the NE content in the kidney of the rats euthanized 8 wk or 12 wk after the surgery (n = 5 per group). The three-way ANOVA + Tukey’s test: *P < 0.0001, denervated groups versus sham groups. NE, norepinephrine.

Successful denervation was confirmed by the observation that renal NE content was ≈72–80% lower in BRDx animals compared with sham-operated (SHAM) in both rat series (*P* < 0.0001), independently of the diet regimen (Fig 1).

**Figure 2.**
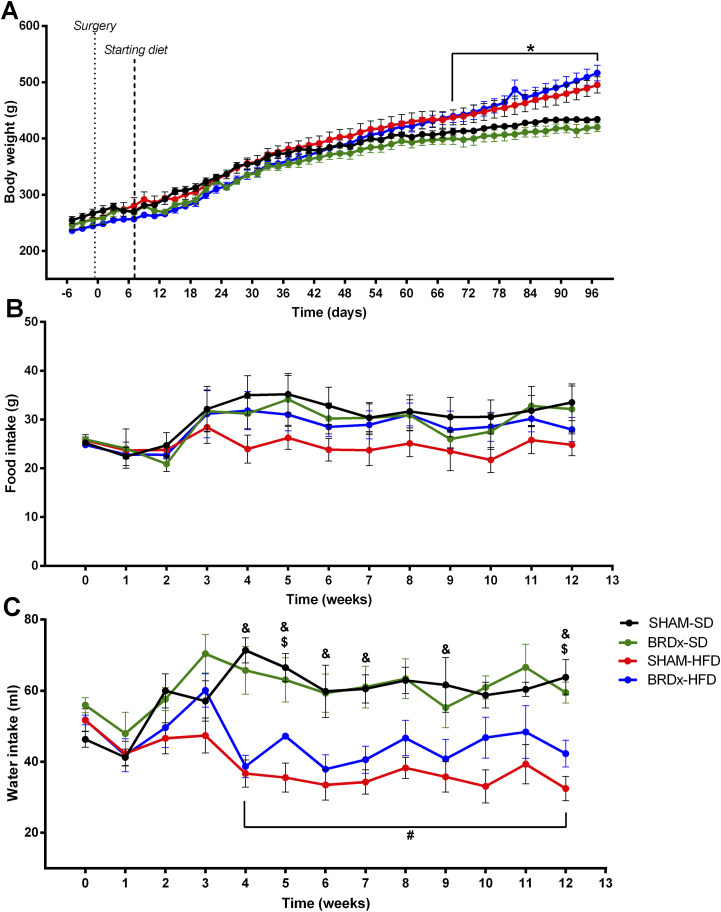
HFD increases BW and decreases WI in both intact and denervated rats. BW (A), FI (B), and WI (C) for each group for the entire protocol. The dotted line represents the surgery day, whereas the discontinuous one is the starting diet day (n = 6 for all groups). The overall repeated-measures MANOVA indicated time effects (*P* < 0.0001) from day 68 in BW and from week 4 in the WI to the end, and time*group interaction effects (*P* < 0.001) at the same points. Repeated-measures MANOVA by group: (*P* from 0.013 to 0.004) day 0 versus day 24 onward, all groups in BW, (*P* from 0.005 to 0.001) week 0 versus week 4 onward, all groups except for BRDx-HFD in WI. Two-way ANOVA + Tukey’s test: *P from 0.0457 to 0.0005, SHAM-HFD versus all groups; #*P* from 0.001 to 0.0007, SHAM-HFD versus SHAM-SD and BRDx-SD; &*P* from 0.015 to 0.0014, BRDx-HFD versus SHAM-SD and BRDx-SD; $*P* from 0.0411 to 0.0402, BRDx-HFD versus SHAM-SD, BRDx-SD, and BRDx-HFD. BRDx, bilateral renal denervation; HFD, high-fat diet.

The overall analysis of the body weight (BW) (Fig 2A), as well as the one by group, indicated an effect of time (*P* < 0.0001) since day 24. HFD intake promoted a significant BW increase with respect to standard diet (SD)–fed groups, independently from BRDx (group*time interaction, *P* = 0.001) from day 68 onward (*P* from 0.0457 to 0.0005). No changes were observed in the average amount of food consumed per week (Fig 2B). Although there was no effect of time on the water intake (WI) (Fig 2C), this was decreased by the HFD when compared to SD-fed groups from week 4 (*P* = 0.001). At weeks 10 and 11, denervation seemed to restore WI in animals that underwent BRDx and then fed with a HFD (BRDx-HFD); however, it was not so at the end of the 12 wk.

**Figure S1.**
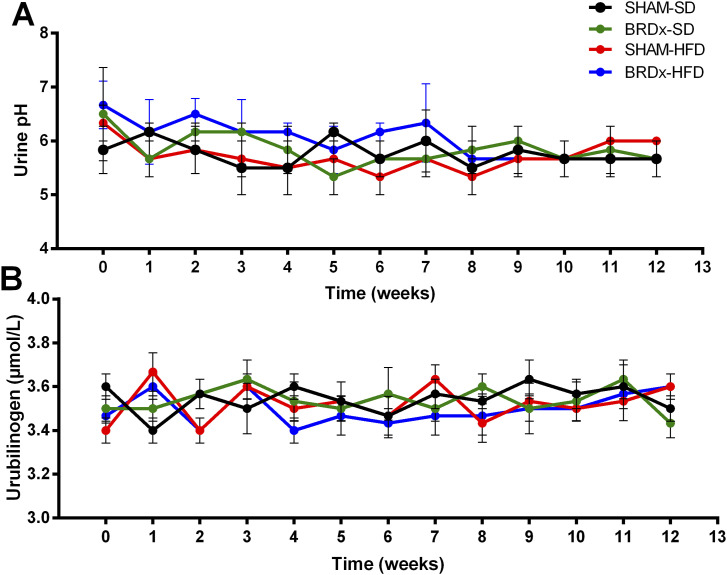
Neither BRDx nor the HFD affects urine pH (A) and urine urobilinogen concentration (B). BRDx, bilateral renal denervation; HFD, high-fat diet.

**Figure S2.**
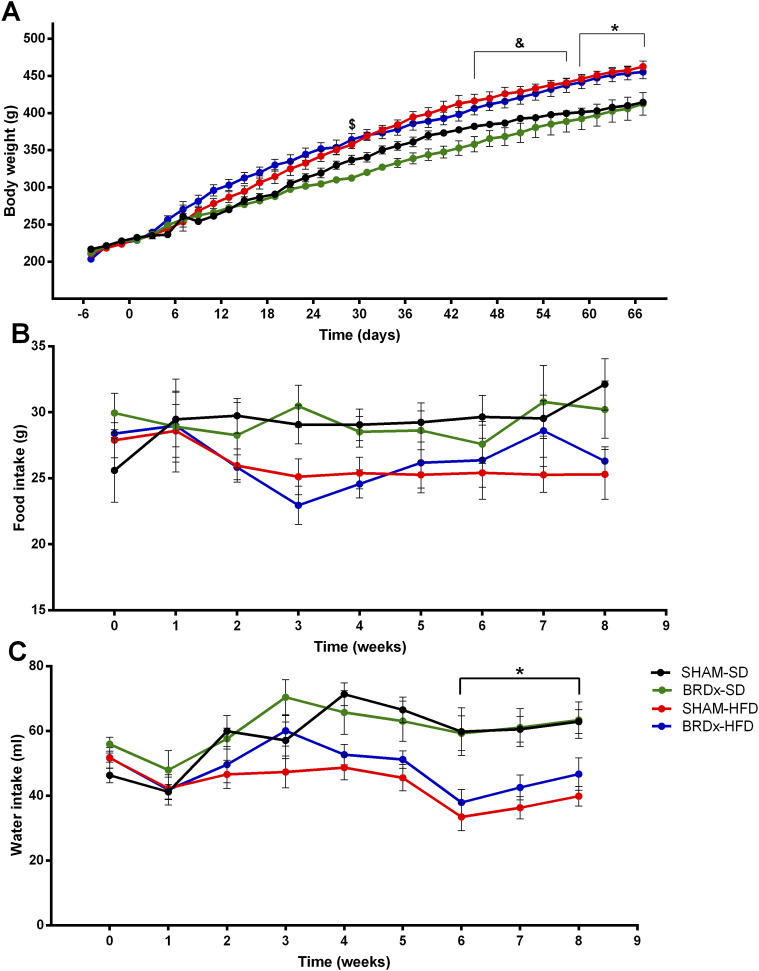
BW (A), FI (B), and WI (C) for each group for the 8-wk series. The overall repeated-measures MANOVA indicated time effects (*P* < 0.0001) from day 36 in BW and from week 6 in the WI to the end, and time*group interaction effects (*P* < 0.001) at the same points. Repeated-measures MANOVA by group: (*P* from 0.019 to 0.005) day 0 versus day 18 onward, all groups in BW, (*P* from 0.003 to 0.001) week 0 versus week 6 onward, all groups in WI. Two-way ANOVA + Tukey’s test: *P from 0.04 to 0.0001, SHAM-HFD and BRDx-HFD versus SHAM-SD and BRDx-SD; &*P* from 0.001 to 0.0007, SHAM-HFD versus BRDx-SD; $*P* = 0.002, BRDx-SD versus all groups. BRDx, bilateral renal denervation; HFD, high-fat diet.

The results obtained from the 8-wk series are represented in Figs S1 and S2.

### Effect of HFD and BRDx on fat depots and leptin, triglycerides, and HDL-cholesterol serum concentrations

**Figure 3.**
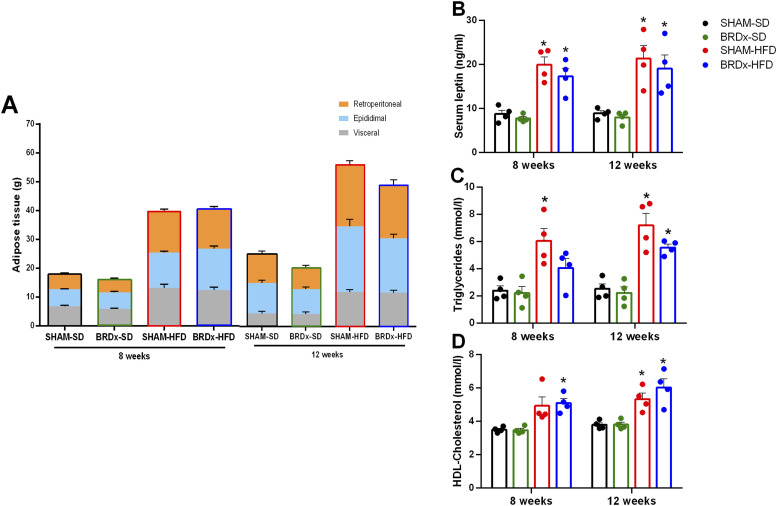
BRDx does not prevent fat accumulation, hyperleptinemia, and dyslipidemia promoted by the HFD. Adipose tissue at 8 wk and 12 wk (A) (n = 6 per group), serum leptin (B), serum TG (C), and HDL-cholesterol (D) (n = 4 per group). Two and Three-way ANOVA + Tukey’s test (adipose tissue and serum parameters, respectively): *P from 0.0492 to <0.0001, SHAM-HFD and BRDx-HFD versus SHAM-SD and BRDx-SD in the same week. BRDx, bilateral renal denervation; HFD, high-fat diet.

Fig 3A shows the amount of visceral, epididymal, and retroperitoneal adipose tissue extracted from animals of the 8-wk series, where HFD produces a significant increase (*P* < 0.0001) compared with the SD, and it is not affected by BRDx. This trend repeated in the 12-wk series (*P* < 0.0001), where HFD increases by ∼ 95% of the values observed at 8 wk. Consistently, blood leptin (Fig 3B), triglycerides (TG) (Fig 3C), and high-density lipoprotein cholesterol (HDL-cholesterol) (Fig 3D) concentrations were up-regulated by HFD (*P* < 0.0001 in all cases) compared with the SD-fed groups. Renal denervation did not affect fat mass, leptin, and HDL-cholesterol in both animals that underwent BRDx and then fed with a SD (BRDx-SD) and BRDx-HFD. Interestingly, TG concentration was significantly lower in BRDx-HFD compared with animals that underwent sham surgery and then fed with a HFD (SHAM-HFD) at 8 wk, but not at 12 wk.

### Effect of HFD and BRDx on blood glucose and insulin

**Figure 4.**
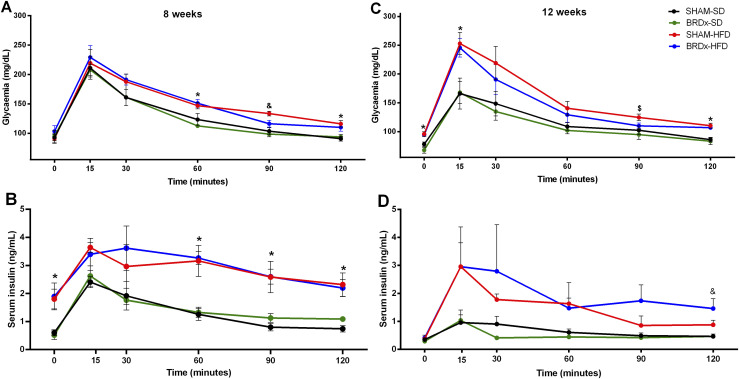
BRDx does not prevent glucose intolerance and insulin resistance promoted by the HFD. Glycemia at 8 (A) and 12 (C) wk and serum insulin at 8 (B) and 12 (D) wk (n = 4 per group). The overall repeated-measures MANOVA indicated time effects (*P* from 0.033 to <0.0001) in all groups and no time*group interaction effects. repeated-measures MANOVA by group: (*P* from 0.0303 to 0.01) minute 0 versus minute 15 onward, all groups. Two-way ANOVA + Tukey’s test: *P from 0.0492 to 0.005, SHAM-HFD and BRDx-HFD versus SHAM-SD and BRDx-SD; &*P* from 0.020 to 0.003, SHAM-HFD versus all groups; $*P* from 0.02 to 0.003, SHAM-HFD versus SHAM-SD and BRDx-SD; &*P* = 0.0402 BRDx-HFD versus SHAM-SD and BRDx-SD. BRDx, bilateral renal denervation; HFD, high-fat diet.

No difference in fasting glucose was found in the 8-wk series (Fig 4A); however, it was significantly increased in both HFD-fed groups at 12 wk (Fig 4C) (*P* < 0.0011). In the GTT, the overall multivariate ANOVA (MANOVA) indicated an effect of time (*P* < 0.0001), group (*P* = 0.0056), and group*time interaction (*P* = 0.0394) in both the 8-wk and 12-wk series. The analysis by time point showed that glycemia was significantly higher at 60, 90, and 120 min after glucose infusion in the SHAM-HFD of the 8-wk series compared with SD-fed animals. BRDx-HFD showed the same pattern, except at 90 min, when glycemia was significantly lower than in BRDx-HFD. In the SHAM-HFD of the 12-wk series, glycemia was significantly increased at 15, 90, and 120 min after glucose infusion in comparison with SD groups; the response was similar in the BRDx-HFD, again with the exception at 90 min, when glucose level was significantly lower than in the SHAM-HFD.

Concerning insulin levels (Fig 4B), the global MANOVA showed an effect of group (*P* = 0.0057), time (*P* < 0.0001), and group*time interaction (*P* = 0.0057). Analysis by time point indicated that insulin levels were up-regulated by HFD (*P* = 0.0072) at 60, 90, and 120 min in the 8-wk series and were not affected by BRDx. In the 12-wk series (Fig 4D), the overall MANOVA only showed an effect of time (*P* = 0.0019) but not of group nor group*time interaction, whereas analysis by time point indicated that the insulin level of the BRDx-HFD was significantly higher (*P* = 0.0234) compared with the SD-fed groups only 120 min after glucose infusion.

### Effect of BRDx and HFD on blood pressure and serum ANG-II

**Figure 5.**
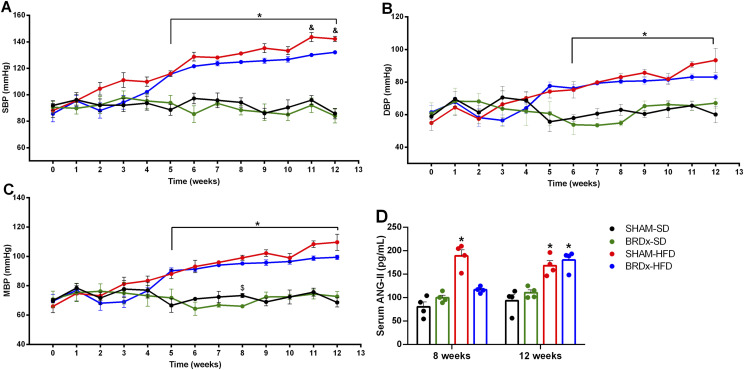
BRDx does not prevent the blood pressure and serum ANG-II increase promoted by the HFD. SBP (A), DBP (B), MBP (C) measured for 12 wk (n = 6 all groups), and serum ANG-II (D). The overall RM-MANOVA indicated time effects (*P* < 0.0001) from week 5 to the end of all groups in SBP and MBP, and from week 6 in DBP; and time*group interaction effects (*P* < 0.0001) in the same points. Repeated-measures MANOVA by group: in SBP (*P* from 0.003 to 0.0002) from week 3 onward in SHAM-HFD and from week 5 in BRDx-HFD; in DBP and MBP (*P* from 0.004 to 0.0001) from week onward in SHAM-HFD and BRDx-HFD. Two-way ANOVA + Tukey’s test: *P from 0.0057 to 0.0001, SHAM-HFD and BRDx-HFD versus SHAM-SD and BRDx-SD; &*P* from 0.0165 to 0.001, SHAM-HFD versus BRDx-HFD; $*P* = 0.0206, SHAM-SD versus BRDx-SD. BRDx, bilateral renal denervation; HFD, high-fat diet; MBP, mean blood pressure.

For the weekly systolic blood pressure (SBP) values (Fig 5A), the overall MANOVA indicated a time effect (*P* < 0.0001) from week 3 onward, group effect (*P* < 0.0001), and group*time interaction (*P* < 0.0001). The HFD significantly increased SBP compared with the SD groups (*P* = 0.0089) since week 5. The SBP of BRDx-HFD was significantly lower than that of SHAM-HFD (*P* = 0.0197) during weeks 11 and 12, although the BRDx-HFD rats still were hypertensive. For diastolic blood pressure (DBP) (Fig 5B), the overall MANOVA showed an effect of time (*P* < 0.0001), group (*P* < 0.0001), and group*time interaction (*P* = 0.0058). The DBP was significantly higher in SHAM-HFD and BRDx-HFD compared with the SD-fed groups (*P* = 0.0025), and no difference compared with BRDx was found. Mean blood pressure (Fig 5C) showed the same pattern as DBP from week 5, with the overall MANOVA indicating the same effects (*P* from 0.0011 to <0.0001).

The quantification of plasma ANG-II at the end of each series (Fig 5D) showed that BRDx attenuated the HFD-induced increase at 8 wk (*P* = 0.003) but not at 12 wk.

### Effect of BRDx and HFD on renal physiology

**Figure 6.**
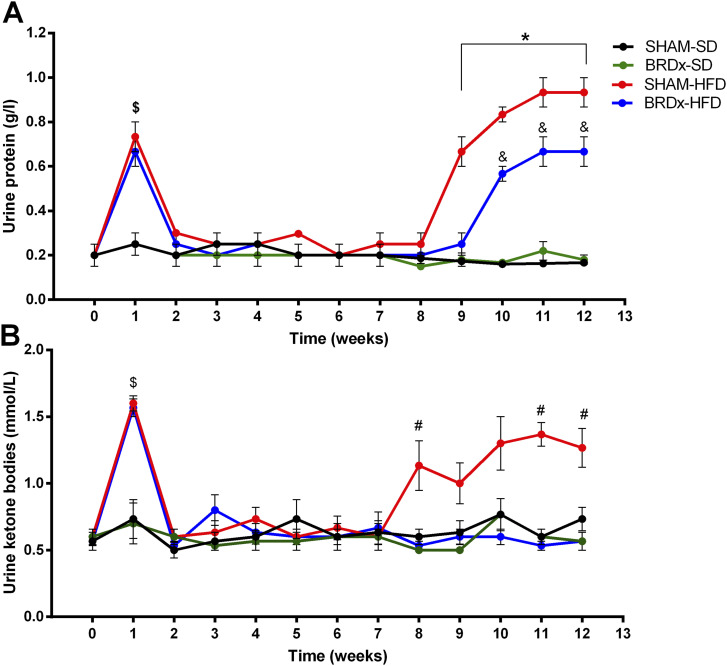
BRDx decreases protein and ketone excretion in rats fed with the HFD. Urinary protein (A) and urine KETs (B) were measured weekly for 12 wk (n = 6, all groups). The overall repeated-measures MANOVA indicated time effects (*P* < 0.0001) from week 1 to the end of all groups, and in both variables, and time*group interaction effects (*P* < 0.0001) at the same points. Repeated-measures MANOVA by group: in SBP (*P* from 0.003 to 0.0002) from week 3 onward in SHAM-HFD and from week 5 in BRDx-HFD; in DBP and MBP (*P* from 0.004 to 0.0001) from week onward in SHAM-HFD and BRDx-HFD. Two-way ANOVA + Tukey’s test: *P from 0.0057 to 0.0001, SHAM-HFD and BRDx-HFD versus SHAM-SD and BRDx-SD; #*P* from 0.026 to 0.0001, SHAM-HFD versus all other groups; &*P* from 0.0165 to 0.001, SHAM-HFD versus BRDx-HFD; $*P* = 0.0206, SHAM-SD versus BRDx-SD. BRDx, bilateral renal denervation; HFD, high-fat diet; MBP, mean blood pressure.

In the weekly quantification of urine protein concentration (Fig 6A), the overall MANOVA showed time (*P* < 0.0001), group (*P* < 0.0001), and group*time interaction (*P* < 0.0001) effects. HFD promoted a sharp increase in protein excretion after the first week of the experiment, followed by an equally blunt decrease. No differences were observed between the groups until week 9, when SHAM-HFD and BRDx-HFD displayed a progressive increase in urine protein content. Concerning the SD-fed groups, from week 9 to week 10, respectively (*P* = 0.0001 and *P* < 0.0001), BRDx attenuated this increase, and protein excretion was significantly lower in BRDx-HFD than in HFD-SHAM (*P* = 0.0004).

For urine ketone bodies (KETs) (Fig 6B), the overall MANOVA indicated an effect of time (*P* < 0.0001), group (*P* < 0.0001), and group*time interaction (*P* < 0.0001). HFD caused a significant increase in ketone excretion with respect to SD-fed groups (*P* = 0.0005) at week 2, independently of BRDx. Nevertheless, a long-term increase in urine KETs was detected in SHAM-HFD (*P* = 0.0059), which BRDx-HFD severely attenuated.

**Figure 7.**
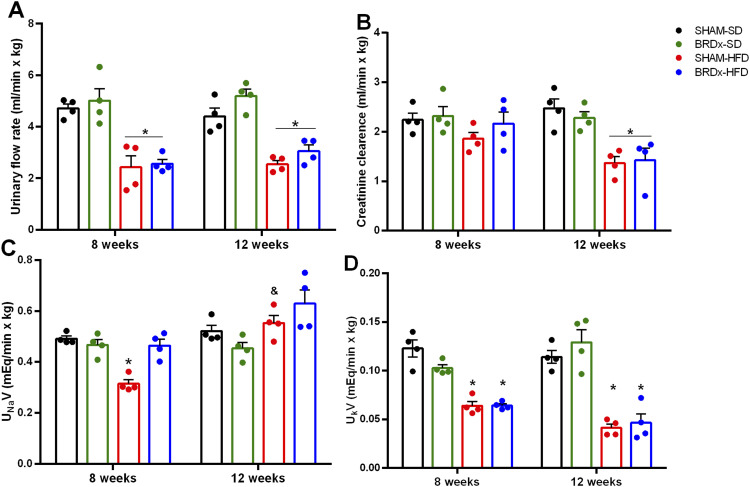
BRDx does not prevent the decrease in UFR and creatinine clearance promoted by the HFD. UFR (A), creatinine clearance (B), UNaV (C), and UKV (D) (n = 4 per group). Three-way ANOVA + Tukey’s test: *P from 0.0235 to <0.0001, SHAM-HFD and BRDx-HFD versus SHAM-SD and BRDx-SD in the same week; &*P* = 0.0008, SHAM-HFD at 8 wk versus SHAM-HFD at 12 wk. BRDx, bilateral renal denervation; HFD, high-fat diet.

**Figure S3.**
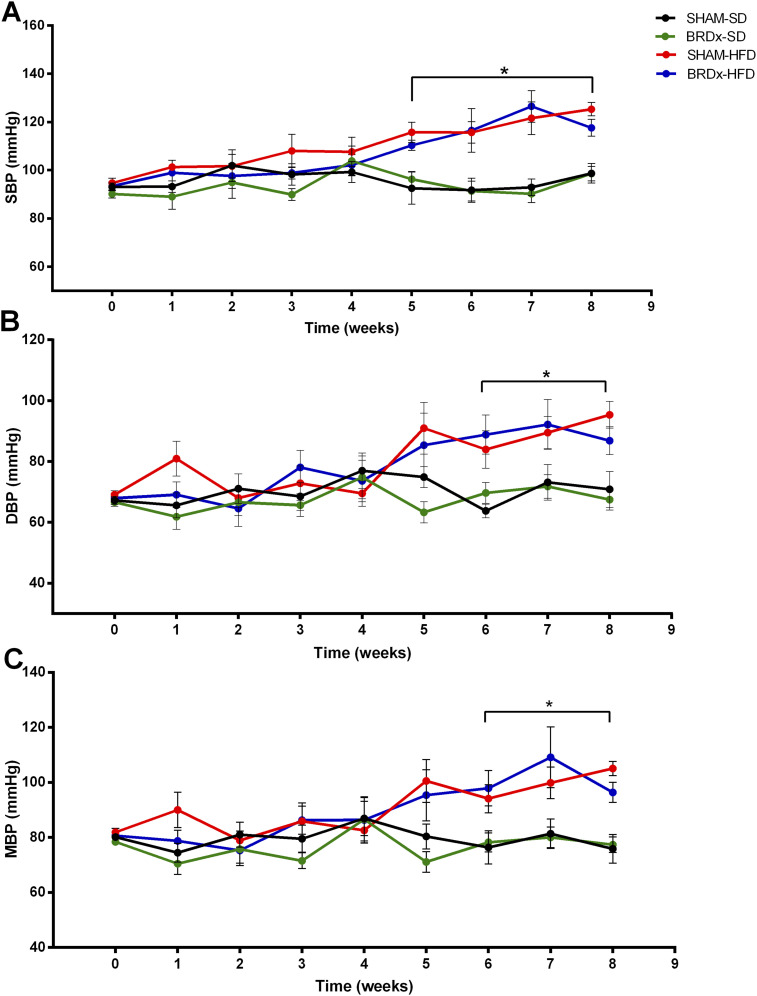
Blood pressure in 8-wk series. SBP (A), DBP (B), and MBP (C) measured for 8 wk (n = 6, all groups). The overall repeated-measures MANOVA indicated time effects (*P* < 0.0001) from week 5 to the end of all groups in SBP and DBP, and from week 6 in MBP; and time*group interaction effects (*P* < 0.0001) in the same points. Two-way ANOVA + Tukey’s test: *P from 0.01 to 0.0001, SHAM-HFD and BRDx-HFD versus SHAM-SD and BRDx-SD. BRDx, bilateral renal denervation; HFD, high-fat diet; MBP, mean blood pressure.

HFD reduces the urinary flow rate (UFR) significantly independently of BRDx (*P* < 0.0001) (Fig 7A) concerning SD-fed rats, in both 8-wk and 12-wk series, whereas creatinine clearance decreased significantly only after 12 wk of HFD (*P* < 0.0001) (Fig 7B). The absolute Na^+^ renal excretion (UNaV) (Fig 7C) significantly decreased in the SHAM-HFD (*P* = 0.0019) with respect to the other groups at week 8, whereas no differences were found in the 12-wk series. There is a statistical increase in Na+ excretion in the BRDx-HFD of the 12-wk series compared with those euthanized at 8 wk (*P* = 0.0003). The HFD significantly decreased absolute K^+^ renal excretion (UKV) (*P* < 0.0001) in both the 8- and 12-wk series (Fig 7D) for SD-fed groups; BRDx elicited no effect. No changes were found in urobilinogen excretion and urine pH (Fig S3).

### Effect of BRDx and HFD on signaling proteins in the kidney

**Figure 8.**
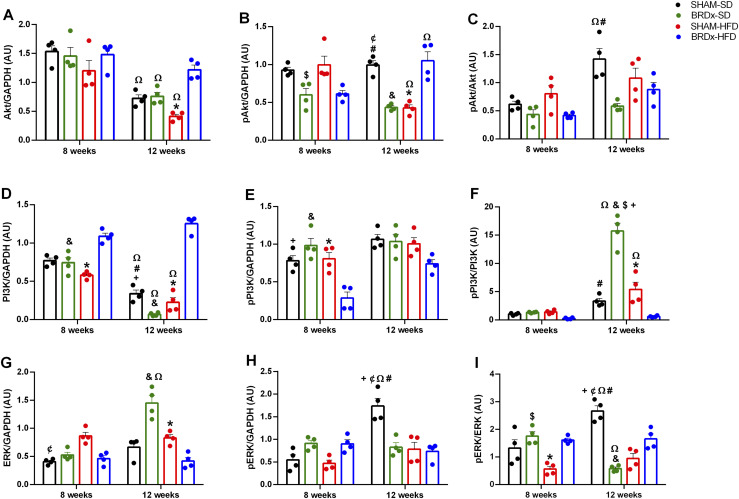
Quantification of the unphosphorylated and phosphorylated AKT, PI3K, and ERK signaling proteins in the kidney. Akt (A), pAkt (B), pAkt/Akt ratio (C), PI3K (D), pPI3K (E), pPI3K/PI3K ratio (F), ERK (G), pERK (H), and pERK/ERK ratio (I) relative expression to GAPDH at the end of 8 and 12 wk (n = 4 per group). #*P* from 0.0084 to <0.0001, SD-SHAM versus SD-BRDx in the same week; ¢*P* < 0.0001, SD-SHAM versus HFD-SHAM in the same week; +*P* from 0.0072 to <0.0001, SD-SHAM versus HFD-BRDx in the same week; $*P* from 0.0012 to <0.0001, SD-BRDx versus HFD-SHAM in the same week; &*P* from 0.0336 to <0.0001, SD-BRDx versus HFD-BRDx in the same week; *P from 0.0493 to <0.0001, HFD-SHAM versus HFD-BRDx in the same week; Ω*P* from 0.0193 to <0.0001, the same condition at 8 versus 12 wk. BRDx, bilateral renal denervation; HFD, high-fat diet.

The renal content of Akt (Fig 8A) and the phospho-Akt (pAkt)/Akt (Fig 8C) ratio did not change in the kidney in the 8-wk series, although a decrease was observed in pAkt (Fig 8B) of the BRDx-SD compared with the SHAM-HFD group (*P* = 0.016). In the 12-wk series, Akt significantly decreases in animals that underwent sham surgery and then fed with a SD (SHAM-SD), BRDx-SD, and SHAM-HFD compared with the 8-wk results (*P* from 0.0020 to 0.0005). Similarly, pAkt decreased in BRDx-SD (*P* = 0.0003) and increased in SHAM-HFD (*P* = 0.0062) in the 8-wk series. In addition, pAkt was down-regulated in BRDx-SD and SHAM-HFD compared with BRDx-HFD (*P* < 0.0001 in both cases). All these changes did not result in significant changes in pAkt/Akt, except that the ratio was higher in SHAM-SD of the 8-wk series compared with the 12-wk series (*P* = 0.0069).

BRDx-HFD displayed a significant increase in the renal content of PI3K (Fig 8D) compared with BRDx-SD and SHAM-HFD (*P* = 0.0005), and PI3K (Fig 8E) compared with the other groups (*P* from 0.0176 to <0.0001), resulting in no changes in the phospho-PI3K p85 (pPI3K)/PI3K ratio (Fig 8F). In SHAM-SD, SHAM-HFD, and BRDx-SD of the 12-wk series, the PI3K content was lower than in the 8-wk series (*P* from 0.0029 to <0.0001), whereas pPI3K increased in BRDx-HFD (*P* < 0.0001). As a result, no significant changes were found in pPI3K/PI3K from the 8-wk series, whereas a significant up-regulation was detected by comparing BRDx-SD (*P* < 0.0001) and SHAM-HFD (*P* = 0.0264) of the 12-wk series. In addition, the increase of pPI3K/PI3K observed in BRDx-SD was significantly different when compared to the SHAM-SD (*P* < 0.0001), SHAM-HFD (*P* < 0.0001), and BRDx-HFD (*P* = 0.0012) group.

**Figure S4.**
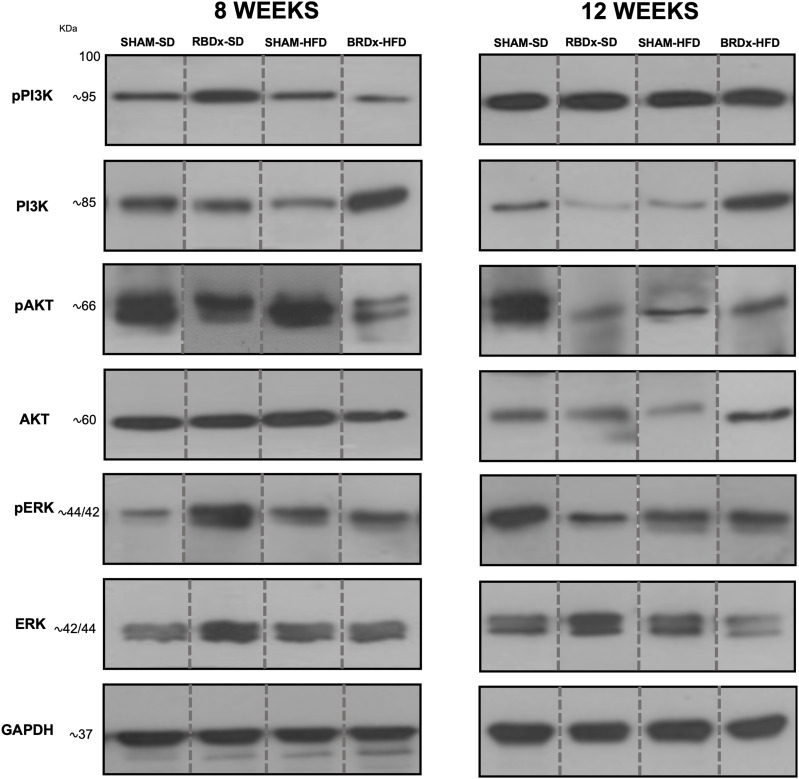
Representative pictures of the Western blot results of unphosphorylated and phosphorylated Akt, PI3K, and ERK signaling proteins in the kidney.

In the 8-wk series, the renal content of p44/42 ERK1/2 (ERK) (Fig 8G) and phospho-p44/42 ERK1/2 (pERK) (Fig 8H) did not show any change between groups, although the ratio resulted in a significant decrease in SHAM-HFD concerning BRDx-SD and BRDx-HFD (*P* = 0.0012 and 0.0048, respectively) (Fig 8I). An upsurge of ERK was observed in BRDx-SD of the 12-wk series compared with the values obtained from the 8-wk series (*P* = 0.0244). The ERK content in BRDx-HFD of the 12-wk series was significantly lower concerning BRDx-SD (*P* = 0.0336) and SHAM-HFD (*P* = 0.0493). The level of pERK was raised in the SHAM-SD when compared to 8 wk (*P* < 0.0001) and was also significantly higher with respect to BRDx-SD, SHAM-HAD, and BRDx-HFD of the same analysis (*P* from 0.0002 to <0.0001). As a result, pERK/ERK increased in the SHAM-SD from the 8- to 12-wk series (*P* = 0.0069), and it was also significantly elevated in BRDx-SD (*P* < 0.0001), SHAM-HFD (*P* < 0.0001), and BRDx-HFD (*P* = 0.0072) of the 12-wk series. Finally, pERK/ERK was significantly lower in BRDx-SD than in BRDx-HFD (*P* = 0.0038). Representative pictures of the Western blots are in Fig S4.

## Discussion

In the present work, we explored the contribution of sympathetic nerve innervation in the onset of renal impairment in a rat model of MetS induced by an HFD in male rats. This study differs from most previous reports because renal denervation was performed before HFD exposure. Under these conditions, our findings show that metabolic and cardiovascular alterations develop largely independently of renal sympathetic input. This provides a mechanistic explanation for the limited long-term efficacy of renal denervation observed in clinical settings and highlights the predominant role of endocrine and metabolic factors—such as leptin, insulin resistance, and RAS activation—in driving disease progression. As expected, the hypercaloric diet promoted a progressive and significant rise in BW (Fig 2A), fat accumulation (Fig 2A and B), and a series of other metabolic, cardiovascular, and renal pathological signs. Surprisingly, these changes were prevented only occasionally by bilateral sympathetic denervation of the kidneys. HFD-fed rats, whether with or without kidney denervation, display almost universally all the signs of MetS: increased circulating leptin levels (Fig 3B) and HDL-cholesterol (Fig 3D), glucose intolerance (Fig 4), and HT (Fig 5A–C). However, the results of BRDx raise several interesting issues that warrant further discussion.

### HFD does not affect the amount of NE released in the kidneys

The NE content within the renal parenchyma was evaluated to assess the efficacy of bilateral kidney denervation, and as illustrated in Fig 1, the surgical procedure was performed successfully. Rodionova et al (2016) demonstrated that renal NE increases by ∼38% 12 wk after denervation. In the present study, we cannot discard that reinnervation occurs; however, the renal NE is significantly lower in all denervated groups compared with the intact ones.

Despite the potential role of RSNA in obesity-related HT and MetS, animals fed with HFD are hypertensive, and blood pressure shows only a slight, nonsignificant difference between obese, intact and denervated animals (Fig 5). It is also important to note that in SHAM rats, HFD does not alter renal NE content compared with a normocaloric SD. All of this aligns with findings in obese, hypertensive humans, who do not significantly increase NE spillover compared with normotensive individuals (Rumantir et al, 1999; Esler et al, 2006). Therefore, RSNA does not completely explain the elevated blood pressure associated with obesity and MetS.

### Long-term HFD promotes kidney impairment, despite the BRDx

The urine pH and urobilinogen content do not change between groups (Fig S1). In contrast, although urine protein and ketone concentration increase (Fig 6A and B), the creatinine clearance decreases (Fig 7B). These data indicate that although the kidneys of the HFD-fed rats can still compensate for the metabolic and cardiovascular challenges, a renal impairment is in progress.

The RSNA appears to have specific effects on renal function. For example, urine protein levels are significantly lower in BRDx-HFD than in SHAM-HFD but still considerably higher than in the STD-fed groups. Proteinuria indicates increased glomerular permeability and, along with decreased creatinine clearance, suggests degeneration of the glomerular structure. This pattern implies that HFD-related factors (such as HT and metabolic changes) promote renal damage, and RSNA worsens this damage despite similar NE levels in SHAM-HFD and SHAM-STD.

One limitation of this study is that BRDx eliminates both afferent and efferent fibers, making it difficult to precisely identify their respective roles. Some observed effects may result from efferent pathways, although the potential influence of afferent signaling cannot be excluded. Afferent sensory fibers containing transient receptor potential vanilloid subtype 1 channels are associated with different processes like the perception of mechanical and chemical stimuli in the kidneys (Stocker & Sullivan, 2023). The removal of these fibers by denervation could alter the kidney’s ability to recognize changes in its own signals, which could explain the observed alterations in renal excretory function (Sullivan et al, 2026). However, because of the complexity of the interactions between neuronal and hormonal factors in this model, the specific role of transient receptor potential vanilloid subtype 1–mediated signaling cannot be established.

### BRDx prevents late ketonuria in rats fed an HFD

Ketonuria is commonly associated with poorly managed diabetes and other conditions characterized by reduced glucose availability (Comstock & Garber, 1990). HFD-fed rats exhibit several risk factors for hyperketonemia and ketonuria. However, the significant increase in renal ketone excretion observed in SHAM-HFD is prevented by BRDx (Fig 6B). This pattern mirrors plasma TG levels, which are significantly higher in SHAM-HFD compared with STD-fed rats and BRDx-HFD in the 8-wk series (Fig 6D). When ketogenesis increases, reabsorption of filtered KETs rises as an adaptive response to prevent the loss of metabolic fuels and NH4+, Na+, and K+ (Palmer & Clegg, 2021). In both HFD-fed groups, we observed transient ketonuria during the first 2 wk of hypercaloric feeding. This suggests that the rat’s metabolism quickly adapts to use KETs as an energy source. Later in the feeding protocol, KET excretion increased in the HFD group, suggesting that ketonuria may depend on saturation of tubular transporters. Very little is known about the molecular, biochemical, and pharmacological regulation of these proteins in rat kidneys. Nevertheless, metabolic acidosis decreases SMCT2 mRNA expression in the mouse nephron (Becker et al, 2010). This indicates that down-regulation of ketone reabsorption in HFD rats might prevent ketoacidosis by increasing excretion. Thus, the drastic reduction of ketonuria in BRDx-HFD suggests that the RSNA prevents renal ketone elimination. This evidence conflicts with the report of Pierre et al (2003), who observed that NE does not affect MCT1 immunoreactivity in cortical culture but up-regulates MCT2. However, De Oliveira et al (2021) reported that BRDx reduces the Na+-glucose cotransporter (SGLT2) expression in the tubular epithelium in a rat model of type 1 diabetes. If so, the increased Na+ concentration in the ultrafiltrate of BRDx-HFD compared with SHAM-BRDx (Fig 7C) might drive KET reabsorption. Regardless of the mechanism by which NE inhibits renal ketone recycling, it is unclear whether bilateral sympathetic denervation might have beneficial or detrimental consequences for the body. Hence, the consequences of decreased ketonuria in BRDx-HFD must be explored carefully.

### HFD decreases WI and urine K+ but does not affect Na+ excretion

In both obese humans and animals, reduced WI has been linked to changes in body fluid distribution and to interactions between diet composition and thirst sensation (Waki et al, 1991; Van Marken Lichtenbelt et al, 1999; Dos-Santos et al, 2022). It is also likely that activation of the hypothalamus–pituitary–epinephrine cascade, promoted by up-regulated RAS, results in salt and water retention that decreases thirst sensation. Regardless of how the rat body manages water distribution under HFD and RSNA conditions, the kidneys respond by decreasing UFR (Fig 7A) and urine K+ levels (Fig 7D). This finding conflicts with the hypothesis that RSNA is the primary driver of water and solute transport in the kidney rather than a participant among other mechanisms. Conversely, Na^+^ excretion decreases in SHAM-HFD animals with respect to the BRDx-HFD after 8 wk of HFD (Fig 7C), whereas no change has been found between SD-fed animals, suggesting that RSNA promotes the Na^+^ reuptake during the early stages of obesity. Moreover, renal denervation prevents ANG-II upsurge in HFD-fed animals (Fig 5D), which likely depends on the dampened catecholamine-stimulated renin release (Hackenthal et al, 1990) and contributes to Na^+^ excretion.

The difference in urine Na^+^ between SHAM-HFD and the other groups gets lost in the 12-wk series, which indicates that in these animals, other factors different from arterial pressure, RSNA, and ANG-II may promote natriuresis.

The reduction of K^+^ excretion observed in the SHAM-HFD rats after 8 wk of the hypercaloric protocol (Fig 7D) may depend on the rise of plasma ANG-II (Fig 5D) and drop of the urine flow (Fig 7A), which counterweight the decrease of water consumption (Fig 2C). However, the amount of excreted K^+^ and water is similar in BRDx-HFD, although these animals do not display any increase in plasma ANG-II. The concentration gradient might drive the K^+^ recycling and transepithelial potential (Palmer et al, 2016), resulting from the boosted water reabsorption in the distal portions of the renal tubules promoted by the antidiuretic hormone (Boone & Deen, 2008).

After 12 wk, the chronic up-regulation of plasma ANG-II in both SHAM-HFD and BRDx-HFD might also inhibit the renal outer medullary K^+^ channels of intercalated cells in the connecting duct, which in turn dampens the electrogenic K^+^ secretion. Furthermore, in HFD-fed rats, the reduced creatinine clearance (Fig 7B) decreases the K^+^ filtration. Because luminal K+ is essential for Na+ reuptake and K+ is only one third of the filtered Na^+^ load, Na^+^ reabsorption likely occurs at the expense of K^+^ excretion.

### BRDx does not prevent the adiposity-related alterations of the MetS

The renal denervation attenuates the HFD-dependent ANG-II uprise in the 8-wk series. However, this adjustment disappears in the 12-wk series (Fig 5D), likely because in obesity and MetS, the increase in the RAS activity depends not only on the canonical, systemic RAS peptide cascade. For example, the expansion of the adipose tissue contributes to about 30% of the circulating levels of angiotensinogen, and in addition, the local, intrarenal RAS results in hyperactivation (Phillips et al, 1993; Giani et al, 2015).

A glance at AT1-R downstream signaling proteins shows that the increase of ANG-II input in the 8-wk series does not affect the pPI3K/PI3K and pAkt/Akt ratio, whereas renal denervation prevents pERK/ERK decrease in HFD-fed animals. This result conflicts with the hypothesis that SHAM-HFD rats should be the most exposed to the harmful effects of the diet because ANG-II–dependent ERK signaling promotes renal inflammation, epithelial–mesenchymal transition, and fibrosis (Mondorf et al, 2000). Nevertheless, the pleiotropic ANG-II intracellular transduction comprises the recruitment of the AT1-R by β-arrestin, paradoxically enhancing ERK signaling and promoting tissular injury (Turu et al, 2019). Supporting this model of “biased signaling,” there is that the pathological response to ANG-II renal infusion, as well as HT, is boosted by selective AT1-R deficiency in the immune system, suggesting that renal ANG-II signaling may have antihypertensive and anti-inflammatory effects (Schellings et al, 2006; Chen et al, 2010; Kendall et al, 2014; Karnik et al, 2015), which, according to the present results, might require RSNA.

### ANG-II intracellular signaling cross-talks with the pathways regulated by leptin and insulin

Leptin displays a significant rise in the plasma of both SHAM- and BRDx-HFD (Fig 3B) that mirrors fat accumulation (Fig 3A and B). Although chronic hyperleptinemia and leptin resistance stimulate the RSNA via hypothalamic and brainstem relays (Haynes et al, 1997; Kuo et al, 2001; Do Carmo et al, 2009; Mark et al, 2009; Dubinion et al, 2011), they also directly disturb kidney homeostasis and harm virtually the whole renal tissue (Korczynska et al, 2021). Hyperphosphorylation of Akt is a marker of renal leptin resistance (Bełtowski et al, 2010) and endothelial dysfunction (Ding et al, 2016). Leptin also promotes mesangial cell hypertrophy via the PI3K and ERK pathways (Lee et al, 2005). However, the results indicate that both the RSNA and diet influence intracellular signaling, and at this stage, it is not possible to fully dissect the relative contribution of each factor. For instance, there is no difference in pPI3K/PI3K between SHAM-SD and SHAM-HFD (Fig 8F) despite leptin being significantly higher in the blood of the latter (Fig 3B), suggesting that hyperleptinemia could not affect this pathway. These pathways represent major convergence points for hormonal mediators altered in obesity and MetS, including ANG-II, leptin, and insulin. Therefore, their analysis was intended to explore whether renal sympathetic input could modulate intracellular responses associated with the metabolic alterations induced by the HFD.

Interestingly, denervation triggers a large increase in pPI3K/PI3K in BRDx-SD relative to SHAM-SD, but a decrease in BRDx-HFD relative to BRDx-SD, indicating that RSNA regulates the PI3K pathway according to the metabolic state. Similar observations regarding insulin and the PI3K-Akt cascade in the 12-wk series can be made. Both RSNA and HFD, alone or in combination, decrease the pERK/ERK ratio (Fig 8F). Hence, NE release in the kidney alters the interplay of RSNA with leptin, insulin, or another metabolic parameter that responds to energy availability and regulates the PI3K pathway and downstream proteins. In the 8-wk series, the systemic insulin resistance observed in HFD-fed animals does not parallel any change in the PI3K-Akt cascade (Fig 8C and F). On the other hand, SHAM-HFD displays a decrease in pERK/ERK, which is reverted in BRDx-HFD. Given the role of the ERK pathway in cell proliferation and growth, renal denervation in obesity and MetS might have detrimental effects on kidney structure and function.

Finally, in the 12-wk series, the PI3K, Akt, and ERK phosphorylated forms abound in SHAM-STD concerning the analog group euthanized after 8 wk (Fig 8C, F, and I), despite there being no change in plasma ANG-II, which suggests the influence of age on the internal renal signaling. It has been suggested that CKD shares some features with aging, which may include the dampened response to oxidative insults and shortening of the cell lifespan promoted by decreased PI3K/Akt and increased ERK signaling, respectively (Terada et al, 2001; Shimamura et al, 2003; Downward, 2004; Slack et al, 2015). Thus, because pAkt/Akt and pERK/ERK (but not pPI3K/PI3K) are significantly higher in SHAM-STD than BRDx-STD, the consequence of renal denervation results is ambiguous.

The present work explores the influence of the autonomic innervation of the kidney on the onset and progression of the MetS. To achieve this, BRDx was performed before the rats were shifted to the HFD, allowing for the observation of overt metabolic and cardiovascular imbalances. The results of the experiments do not contribute to solving the problem of the safety and efficiency of surgery in humans. Still, they merely try to dissect the effects of the neural input to the kidney from the changes in hormonal signaling promoted by the hypercaloric diet. The autonomic renal innervation does not play a pivotal role because obese animals show HT and the early signs of CKD, independently of the surgery, which blames ANG-II, leptin, insulin, and all the other possible players that link the alteration of the metabolic homeostasis with the cardiorenovascular sequelae. On the other hand, the results obtained from the 12-wk series underscored that renal denervation might also have risky effects, as suggested by glucose handling and ketonuria in BRDx-HFD rats. This observation opens the way to additional questions about the neural and hormonal interplay in the pathophysiology of MetS, especially in the kidney.

### Conclusions

This study investigated the role of RSNA in cardiovascular and renal function in male rats with MetS induced by an HFD. Results show that disrupting RSNA in HFD-fed animals does not significantly improve blood pressure or most renal impairment parameters, especially in the long term. For instance, BRDx only delays increased protein excretion in HFD rats. Similarly, the procedure normalizes Na^+^ excretion and prevents the rise in plasma ANG-II caused by HFD at 8 wk, but not at 12 wk. In addition, changes in plasma TG and urine KETs in BRDx-HFD compared with SHAM-HFD suggest RSNA has a specific role in lipid oxidation during positive energy balance. These findings are reinforced by the interaction between RSNA, ANG-II, leptin, and insulin signaling, which varies with feeding state and possibly age. For example, RSNA appears essential to prevent renal disruption caused by ANG-II in 8-wk HFD rats. However, the procedure also increases the pERK/ERK ratio, which may heighten susceptibility to leptin and insulin’s mitogenic and proliferative effects. Further experiments are needed to clarify the neural and hormonal regulation of kidney function and how this organ responds and adapts to metabolic challenges.

## Materials and Methods

### Animals

Juvenile male Wistar rats, weighing 100–120 g (∼23–30 d old), were donated by Dr. Carolina Escobar Briones, head of the Laboratory of Biological Rhythms and Metabolism at the Universidad Nacional Autónoma de México. The animals were housed individually in acrylic cages under a 12/12-h light–dark cycle at 23 ± 1°C, with free access to food (Laboratory Rodent Chow 5001, LabDiet) and filtered water. They were monitored to evaluate their growth until they reached a weight between 200 and 210 g for surgery. The experimental procedures were approved by the Ethical Committee of the Facultad de Ciencias Químicas of Universidad Autónoma de San Luis Potosí (CEID-FCQ, CEID2014030), in strict accordance with the Mexican Guidelines for the Care and Use of Experimental Animals (NOM-062-ZOO-1999) and the European Convention for the Protection of Vertebrate Animals used for Experimental and other Scientific Purposes (Council of Europe No. 123, Strasbourg 1985).

### Experimental protocol

#### Series 1 (8 wk)

The animals were randomly assigned into four groups: SHAM-SD (n = 6) or SHAM-HFD (n = 6), and BRDx-SD (n = 6) or BRDx-HFD (n = 7). The standard laboratory diet (vide supra) contained 4.5% fat and provided 3.36 kcal/g. The HFD was obtained by adding 15% pork fat (HEB; 886 kcal/g) and 15% margarine (Primavera, Upfield; 545 kcal/g) to the SD. According to the nutritional facts indicated by the manufacturers, the HFD contained ∼28.60% of fat and provided ∼217 kcal/g. Animals stayed under general conditions before the surgery (SHAM or BRDx) until they reached or exceeded their presurgery weight; only the rats meeting this requirement were subjected to the feeding protocol until euthanasia. The rats were anesthetized with an overdose of sodium pentobarbital (Pisabental, PiSA Agropecuaria). The renal hilum was occluded, and both kidneys were quickly removed and immediately frozen at −80°C for further analysis. Then, animals were transcardially perfused with 0.9% saline, followed by 4% PFA (Sigma-Aldrich Corp.) diluted in phosphate buffer (0.1 M, pH 7.4). The retroperitoneal, epididymal, and visceral adipose depots were removed for weighing.

#### Series 2 (12 wk)

The animals in series 2 underwent the same procedure as in series 1, except rats had access to their respective diets for 12 wk instead of 8 wk.

### Bilateral renal denervation

Briefly, rats were anesthetized with sodium pentobarbital (50 mg/kg, intraperitoneally, Pisabental, PiSA Agropecuaria) and placed on a thermostatic operating table at 37°C, with general asepsis, and under stereoscope visualization (X25). After the abdominal midline incision, both kidneys were exposed, and the renal vessels were isolated from connective tissue and perirenal fat. After mechanically doffing the visible nerves from the kidney up to the renal aortic ganglion (also removed), the vessels were coated for 2 min with 10% phenol in absolute ethanol. The muscle layers of the abdominal wall were stitched with absorbable suture (Chromic Gut 4-0, COVIDIEN), and the skin was closed using a nonabsorbable suture (SEDA-SILK, 4-0, ETHICON, Johnson & Johnson). Ceftriaxone (1 g/kg, PiSA Farmacéutica) and ketoprofen (0.5 g/kg Profenid, Farmar Health Care Services) were administered after the surgery. Rats were placed in warmed cages until complete recovery from the anesthesia (30–50 min) and then returned to their home cages. In the sham surgery, the renal nerves and ganglia were isolated from the connective tissue but preserved.

### Analytical techniques

Animals from series 1 and 2, and the respective samples obtained before and after euthanasia, were analyzed as follows.

#### BW, food and water consumption, and blood pressure recording

BW, FI, and WI were measured every 48 h. SBP, DBP, and mean blood pressure were assessed weekly, using a noninvasive tail-cuff CODA device (Kent Scientific). Animals were acclimated to the tail-cuff procedure before data collection to reduce stress-related variability. For that, they were maintained in acrylic restrainers, whereas an occlusion cuff was placed at the base of the tail, and a sensor (VPR-cuff) was placed 1 cm from the occlusion cuff. 15 correct BP measures were obtained from each animal. The efficiency of this system provides about 99% correlation with telemetry and direct blood pressure measurement (Fraser et al, 2001; Feng et al, 2008).

#### Plasma metabolic and hormonal parameters

An intraperitoneal glucose tolerance test (GTT) was performed two days after the metabolic cage assays. Rats were fasted for 24 h; the tail was cleaned with 70% ethanol and cut 1 mm from the tip using a sharp, sterile scalpel. Fasting blood glucose (GLU) was measured with a glucometer (Accu-Chek, Roche), and then, 1 g/kg of D-glucose solution (50%, PiSA) was administered intraperitoneally. Blood samples collected before (time 0) and 15, 30, 60, and 120 min after glucose administration were stored in Eppendorf tubes (∼200 μl) containing a clot-activator gel and centrifuged at 1,070 × g for 10 min. These samples were used to determine glucose concentration and stored at −80°C for later insulin (INS) quantification. This was performed using a sandwich ELISA kit (APLCO Immunoassays) following the manufacturer’s instructions and using 30 μl of serum per sample. Blood samples (∼2–3 ml) collected during euthanasia and centrifuged as previously mentioned were used to determine TG and HDL-cholesterol concentration by colorimetric enzymatic kits (Spinreact; Colestat, Weiner Lab), as well as leptin (LEP) and ANG-II by a sandwich ELISA kit (BioVendor; MyBioSource), following the manufacturer’s instructions and using 2.5–50 μl of serum per sample. Results were quantified at 450–500 nm using a spectrophotometer (Novaspec II Visible, Amersham Pharmacia Biotech).

#### Renal NE quantification

To evaluate the effectiveness of the BRDx, the NE content in a kidney from each animal was measured using ELISA (cat no. MBS269993; MyBioSource). Denervation success was defined when tissue NE levels showed significant statistical differences between the denervated and sham groups at the end of the protocol (Muhlbauer et al, 1997).

#### Renal parameters

On the same day of blood pressure measurement, the urine proteins, KETs, urobilinogen content, and pH were quantified by means of a dry chemical method kit (urine strips, URIN-10, Spinreact). To determine renal excretory function, 4 days before the end of each protocol, five rats were randomly selected from each group and placed in metabolic cages with their respective diets to collect 24-h urine and plasma. UFR was estimated as V/T × BW, where V is the urine volume in mL, and T is the time in minutes. Blood samples (0.4–0.5 ml) were collected from the tail vein, put in clot-activator gel tubes (BD Vacutainer 367983, BD), and centrifuged at 4,000 rpm for 10 min. UNaV and UKV were taken as UNa × UFR and UK × UFR, being UNa and UK measured by flame photometry (Flame Photometer Model 943; Instrumentation Laboratory SpA). Creatinine clearance was estimated as follows: creatinine clearance = Ucr × UFR/Pcr, with Ucr and Pcr being urine and plasma creatinine concentrations determined by a colorimetric enzymatic kit (Creatinine-J, Spinreact) at 500 nm with a spectrophotometer (vide supra).

#### Kidney protein extraction and immunoblotting

The whole kidney tissue was placed in an ice-cold lysis buffer solution containing 1 mM Hepes, 150 mM NaCl, 1 mM EGTA, 0.1 mM MgCl2, and 0.5% Triton X-100, and supplemented with a protease and phosphatase inhibitor cocktail (1×, Halt, Thermo Fisher Scientific). The samples were immediately homogenized (Tissue Tearor Homogenizer, BioSpec Products) at 4°C for ∼15 s., and then centrifuged (Thermo MicroCL 21R, Thermo Fisher Scientific) at 21,100 × g for 15 min at 4°C. The protein concentration was determined by the bicinchoninic acid method (Sigma-Aldrich). Kidney protein samples (10–50 μg) were mixed with 2× Laemmli buffer and heated at 95°C for 5 min, then electrophoretically separated on 8–12% SDS–PAGE gels at 120 V/50 mA for 90 min. Separated proteins were transferred by semi-dry transfer apparatus (Trans-Blot SD Cell, Bio-Rad Laboratories) to polyvinylidene difluoride membranes (0.45 μm, Immobilon-P, Millipore). Molecular weight markers (∼10–250 kD; Precision Plus Protein All Blue Standards, Bio-Rad Laboratories) were used to estimate molecular mass. Blots were blocked with 5% bovine serum albumin or 5% nonfat dry milk in Tris-buffered saline (pH 7.6)/0.1% Tween-20 (TBST). Blots were incubated with primary antibodies to Akt (1:1,000, #9272; Cell Signaling), pAkt (Ser473, 1:500, #4060; Cell Signaling), PI3K p85 (1:1,000, #4292; Cell Signaling), pPI3K (Tyr458; 1:1,000, #4228; Cell Signaling), ERK (1:1,000, #9102; Cell Signaling), and pERK (Thr202/Tyr204, 1:1,000, #4370; Cell Signaling), and loading control protein GAPDH (1:4,000, sc-25778; Santa Cruz Biotechnology Inc.) at 4°C, overnight. Blots were washed with TBST and then exposed to the secondary antibody conjugated to horseradish peroxidase (1:5,000, sc-2004; Santa Cruz Biotechnology Inc.) diluted in TBST/5% bovine serum albumin or 5% nonfat dry milk at 25°C for 90 min. Detection of specific proteins was accomplished using an enhanced chemiluminescence kit (Immobilon Western; Millipore); according to the manufacturer’s instructions, blots were exposed to a X-ray film (The Number 1 Dental Film, Carestream Dental).

#### Experimental design

The animals were randomly assigned to their respective group using R software (V3.4) when they reached a BW between 225 and 255 g. Following the elimination criteria, excluded rats were replaced with others, who were assigned sequentially as others were eliminated (a random process) using a spiral tracking strategy until the groups were completed. The number of animals used in each analysis is indicated in the corresponding figure legends, because some measurements required specific biological samples, and certain analyses were performed in subsets of animals from each experimental group. For some analytical determinations, only small blood volumes were collected to minimize physiological stress. Because the study’s primary outcome was BP in both series, it was a three-way, completely randomized, longitudinal study (Cook & Ware, 1983). The three approaches were denervation, a diet with two levels, and time with repeated measurements. The BP, BW, FI, WI, and urine parameters (PROT, KET, URI, and pH) were measured weekly, on the same day. In contrast, the amount of fat depots, blood metabolic parameters (LEP, ANG-II, INS, GLU, TRY, and HDL), renal hemodynamic parameters (UFR, creatinine clearance, UNaV, UKV), and signaling proteins (Akt, pAkt, PI3K, pPI3K, ERK, pERK) were cross-sectionally measured at the end of each protocol. Densitometric results were reported as integrated band areas (band density) and expressed as the ratio of the target protein to the loading control (GAPDH) using ImageJ software (NIH, USA).

### Statistical analysis

The overall time course of each continuous variable (BP, BW, FI, WI, GTT, Insulin, PROT, KET, URI, and pH) was analyzed (95% confidence interval) using three-way repeated measures of MANOVA (Weinfurt, 2000), one-way measure being the diet, another the denervation, and the other time, with repeated measurements, looking for time, group, and/or time*group interaction effects. To assess the effect of time within each group, a repeated-measures ANOVA was performed, with no assumption of sphericity; the Geisser–Greenhouse correction was used instead. The response variables were modeled by linear fixed models (regression analysis, one-way, two-way, and three-way ANOVA), their interactions, and mixed effects with Geisser–Greenhouse correction; after testing the residuals for normality (Shapiro–Wilk test) and homoskedasticity (Brown–Forsythe test) (Heiberger & Holland, 2004), the best models were chosen based on the highest r^2^ (determination coefficient = explained variation). The lowest Akaike information criterion score (Akaike, 1974) and the parsimony principle were also considered. Post hoc multiple comparisons and power analysis were conducted using a Tukey test and G*Power. The alpha level was set at 0.05. Statistical analysis was performed using JMP V10 (SAS Institute) and GraphPad Prism V9 (GraphPad Software); all values are represented as the mean ± SEM, and estimates are reported with 95% confidence intervals.

## Supplementary Material

Reviewer comments

## Data Availability

The data that support the findings of this study are available from the corresponding author upon reasonable request.
