# Supplementary material for: The onset of hypertension in metabolic syndrome is independent of renal sympathetic innervation
Source: Life Sci Alliance. 2026 Jul 14;9(10):e202503568. doi: 10.26508/lsa.202503568 (PMC13370070; doi:10.26508/lsa.202503568)
Supplement: Reviewer comments [file LSA-2025-03568_review_history.pdf]

The onset of hypertension in metabolic syndrome is independent of renal sympathetic innervation

Omar Flores-Sandoval, Skarleth Cardenas-Romero, Adrián Báez-Ruiz, Roberto Carlos Salgado-Delgado and Nadia Saderi
DOI: https://doi.org/10.26508/lsa.202503568

Corresponding author(s): Dr. Nadia Saderi (Autonomous University of San Luis Potosí)

Review Timeline:

Submission Date:	2025-11-11
Editorial Decision:	2026-02-06
Revision Received:	2026-05-22
Editorial Decision:	2026-06-16
Revision Received:	2026-06-26
Accepted:	2026-06-29

Scientific Editor: Tim Fessenden

Transaction Report:

(Note: With the exception of the correction of typographical or spelling errors that could be a source of ambiguity, letters and reports are not edited. The original formatting of letters and referee reports may not be reflected in this compilation.)

February 6, 2026

Re: Life Science Alliance manuscript #LSA-2025-03568-T

Dr. Nadia Saderi
Autonomous University of San Luis Potosí
Laboratorio de Neuroanatomía Funcional, Facultad de Ciencias
Av. Parque Chapultepec 1570, Privadas del Pedregal
San Luis Potosí, San Luis Potosí 78295
Mexico

Dear Dr. Saderi,

Thank you for submitting your manuscript entitled "The onset of hypertension in metabolic syndrome is independent of renal sympathetic innervation in male rats" to Life Science Alliance. The manuscript was assessed by expert reviewers, whose comments are appended to this letter. We regret the delay in returning a decision to you, due to reviewer unavailability, and we sincerely appreciate your patience.

As you will see, both reviewers appreciate the several cardiovascular parameters reported here and the impact of renal denervation on them, finding potential value of these results for the field. Reviewer 1 however notes important details that should be included, on replicates and on methodology, and requests clarifications in the text to motivate this study and frame the advance it provides. Reviewer 2 appreciates these findings and makes several suggestions to improve the text, as well as requests to remark on potential limitations. We invite you to submit a revised manuscript addressing these reviewers' comments.

I would be happy to discuss the revision in more detail via email or phone/videoconferencing. Please let me know which option you prefer, if any.

To upload the revised version of your manuscript, please log in to your account: https://lsa.msubmit.net/cgi-bin/main.plex

You will be guided to complete the submission of your revised manuscript and to fill in all necessary information. Please get in touch in case you do not know or remember your login name.

The typical timeframe for revisions is three months. Please note that papers are generally considered through only one revision cycle, so strong support from the referees on the revised version is needed for acceptance.

While you are revising your manuscript, please also attend to the below editorial points to help expedite the publication of your manuscript. Please direct any editorial questions to the journal office. When submitting the revision, please include a letter addressing the reviewers' comments point by point.

Thank you for this interesting contribution to Life Science Alliance. We hope that the comments below will prove constructive as your work progresses, and we are looking forward to receiving your revised manuscript.

Sincerely,

Tim Fessenden
Scientific Editor
Life Science Alliance

A. THESE ITEMS ARE REQUIRED FOR REVISIONS

-- A letter addressing the reviewers' comments point by point.

-- An editable version of the final text (.DOC or .DOCX) is needed for copyediting (no PDFs).

-- High-resolution figure, supplementary figure and video files uploaded as individual files: See our detailed guidelines for preparing your production-ready images, https://www.life-science-alliance.org/authors

-- Summary blurb (enter in submission system): A short text summarizing in a single sentence the study (max. 200 characters

including spaces). This text is used in conjunction with the titles of papers, hence should be informative and complementary to the title and running title. It should describe the context and significance of the findings for a general readership; it should be written in the present tense and refer to the work in the third person. Author names should not be mentioned.

-- By submitting a revision, you attest that you are aware of our payment policies found here: https://www.life-science-alliance.org/copyright-license-fee

B. MANUSCRIPT ORGANIZATION AND FORMATTING:

Full guidelines are available on our Instructions for Authors page, https://www.life-science-alliance.org/authors

We encourage our authors to provide original source data, particularly uncropped/-processed electrophoretic blots and spreadsheets for the main figures of the manuscript. If you would like to add source data, we would welcome one PDF/Excel-file per figure for this information. These files will be linked as supplementary "Source Data" files.

IMPORTANT: It is Life Science Alliance policy that if requested, original data images must be made available. Failure to provide original images upon request will result in unavoidable delays in publication. Please ensure that you have access to all original microscopy and blot data images before submitting your revision.

Reviewer #1 (Comments to the Authors (Required)):

Summary: This manuscript demonstrates that bilateral renal denervation does not significantly improve blood pressure or most renal impairment parameters, especially in the long term, of male Wistar rats fed with a high-fat diet. Although the authors assess a wide range of parameters, a major concern is the lack of a clear rationale for some of the experiments performed, as well as the limited innovation of the study.
Major points:
1. Methods: The methods section require substantially more detail. The authors should clearly state the number of rats used in each experiment, the age at which animals underwent surgery, and other relevant procedural details. In addition, the source of "donated rats" should still be explicitly stated.
2. The number of rats appears to differ across experiments based on the figure legend. It is unclear whether different cohorts of animals were used for different experiments. If so, the efficiency of renal denervation of all rats should be presented. If not, the authors should explain why the reported sample size differ between experiments.
3. The rationale for examining both 8-week and 12-week high-fat diet interventions is unclear. If the intent is to compare short-term vs long-term effects, these time points are relatively close. A shorter duration (e.g., 10 days) may be more appropriate for assessing acute effects and would strengthen the experiment design.
4. The manuscript would benefit from substantial shortening, particularly a more concise and focused Discussion.
5. Numerous studies have already examined the effects of renal denervation on high-fat diet-induced pathophysiology. The authors should more clearly articulate the novelty of the present study and specify how their findings advance the existing literature.

Reviewer #2 (Comments to the Authors (Required)):

The manuscript by Flores-Sandoval and colleagues addresses an important topic with clear translational relevance, namely the autonomic, cardiovascular, metabolic, and renal consequences of chronic exposure to a high-fat diet (HFD). The study provides several noteworthy findings. First, HFD induces a progressive and sustained mild hypertension, affecting systolic, diastolic, and mean blood pressure. Second, renal denervation exerts a modest and transient effect, primarily influencing systolic blood pressure and circulating angiotensin II levels during intermediate stages. Third, with prolonged exposure, the effects of a chronic hypercaloric diet appear to override autonomic modulation, consistent with the progression of metabolic syndrome.
In addition, the data support the concept that dietary fat intake is the predominant driver of adiposity and metabolic disturbances. Renal denervation shows limited and short-lived metabolic effects, particularly with respect to triglyceride regulation. Overall, the findings reinforce the broader conclusion that autonomic modulation plays a relatively minor role in long-term metabolic outcomes under sustained HFD conditions.
The manuscript is well presented, and the results are interesting and generally well supported. Nevertheless, several concerns should be addressed to strengthen the conclusions:
1. The authors state that renal sympathetic activity was evaluated; however, sympathetic nerve activity was not directly recorded. Therefore, it should be clarified that the study assesses the effects of total renal denervation on the measured outcomes, rather than direct measurements of renal sympathetic nerve activity (RSNA).
2. The renal nerve contains both efferent and afferent fibers. The manuscript would benefit from a discussion of the relative contribution of afferent versus efferent signaling to the observed effects. In this context, the potential role of TRPV1-expressing afferent fibers in mediating cardiovascular and renal responses should be addressed.

3. Renal denervation was assessed by measuring noradrenaline content in renal tissue. While this is an accepted approach, complementary evaluation of tyrosine hydroxylase expression would strengthen the validation of effective denervation.
4. Blood pressure was measured using an indirect tail-cuff method, which has known limitations in accuracy and sensitivity in rodents. The authors should discuss whether the observed effects would be expected to persist if blood pressure were measured using direct intra-arterial recordings.
5. The manuscript reports a lower water intake in the standard diet (SD) group. A mechanistic explanation for this finding should be provided or discussed.
6. Renal denervation reduced circulating angiotensin II levels in HFD-fed animals at 8 weeks, but not at 12 weeks. How do the authors explain this temporal difference? Could renal nerve reinnervation or compensatory activation of other components of the renin-angiotensin system account for this effect?
7. Given that high-fat intake itself can directly promote renal injury, the authors should clarify how they distinguish the contributions of renal nerves and/or angiotensin II from the direct effects of HFD on blood pressure, metabolic parameters, renal function, and intracellular signaling pathways (Akt, PI3K, and ERK1/2).

Reviewer #1 (Comments to the Authors (Required)):

Summary: This manuscript demonstrates that bilateral renal denervation does not significantly improve blood pressure or most renal impairment parameters, especially in the long term, of male Wistar rats fed with a high-fat diet. Although the authors assess a wide range of parameters, a major concern is the lack of a clear rationale for some of the experiments performed, as well as the limited innovation of the study.

Major points:

1. Methods: The methods section require substantially more detail. The authors should clearly state the number of rats used in each experiment, the age at which animals underwent surgery, and other relevant procedural details. In addition, the source of "donated rats" should still be explicitly stated.

REPLY:

We thank the Reviewer for these observations. Indeed, the group size has been indicated only in the figure legend and not in the main text. This information has been incorporated into the corresponding sections in the Materials and Methods section specifically in the experimental design.

The animals underwent surgery approximatively on the fifth week of their lives. Specifically, after weaning (postnatal day 23), rats were placed in their home cages and, after a week of acclimatation, were assigned to the experimental protocol. The age rats at the beginning of the experiment are now specified in the main text. Nevertheless, rats underwent surgery only when their weight was between 200g and no more than 210g.

The animals were provided free of charge by Dr. Carolina Escobar Briones and bred in the School of Medicine vivarium at Universidad Autónoma de México. Dr. Escobar is now mentioned in the main text.

2. The number of rats appears to differ across experiments based on the figure legend. It is unclear whether different cohorts of animals were used for different experiments. If so, the efficiency of renal denervation of all rats should be presented. If not, the authors should explain why the reported sample size differ between experiments.

REPLY:

We thank the Reviewer for this observation. With respect to renal denervation, the noradrenaline quantification data describe the results obtained by the whole set of rats that

underwent surgery. The variations in sample size observed in some experiments indicate that different measurements required specific biological samples (e.g., kidney tissue, urine, or serum) and were therefore performed on subsets of animals belonging to each experimental group. In addition, for some biochemical determinations, small blood volumes were collected to minimize the physiological stress associated with testing in metabolic cages shortly before sacrifice. This clarification has been added to the Materials and Methods section.

3. The rationale for examining both 8-week and 12-week high-fat diet interventions is unclear. If the intent is to compare short-term vs long-term effects, these time points are relatively close. A shorter duration (e.g., 10 days) may be more appropriate for assessing acute effects and would strengthen the experiment design.

REPLY:

The starting point for this study was to analyze how long does high-fat diet takes to promote the canonical hallmarks of the metabolic syndrome. For that, we preferred the chronic exposure to the metabolic challenge, which might be a better representation of the human obesity-associated hypertension and increased cardiovascular risk. Rats were fed with the hypercaloric diet when juvenile (days 37-42) and throughout their emerging (8 weeks) and young (12 weeks) adulthood [we refer the Reviewer to some general reviews, such as Ghasemi et al., XCLI J. 2021 (https://www.excli.de/index.php/excli/article/view/4072)]. Moreover, previous studies in our lab showed that ectopic fat accumulation, dyslipidemia, and hypertension require at least 8 weeks of high-fat diet to be statistically significant. Similar observations have also been reported by several Authors, for example: Kovacs and Hajnal, Chem Senses. 2022 (https://doi.org/10.1093/chemse/bjac020); Di Majo et al., Antioxidants (Basel). 2022;12(1):89. (doi:10.3390/antiox12010089). Thus, the additional 12-week group was also necessary to assess the chronic nature of the metabolic deregulation and hypertension.

It has been stated in the main text that rats underwent the experiment from their juvenile age, rather than in their young adulthood.

4. The manuscript would benefit from substantial shortening, particularly a more concise and focused Discussion.

REPLY:

We agree with the Reviewer's opinion, and we have made an effort to reduce the manuscript length. Nevertheless, we would not like to exclude any of the results that deserve a discussion, especially those that are controversial or require an integrative interpretation.

5. Numerous studies have already examined the effects of renal denervation on high-fat diet-induced pathophysiology. The authors should more clearly articulate the novelty of the present study and specify how their findings advance the existing literature.

REPLY:

We thank the Reviewer for highlighting this point. We agree that there is a huge number of reports that employed the same animal model and that evaluate the effect of renal denervation in both humans and animals. However, the present study investigates how much renal sympathetic input contributes to the cardiovascular complications of obesity. For that, rats were denervated before the exposure to the high-fat diet (and not when already obese and hypertensive). This has been stated in the last sentence of the Introduction and the first paragraph of the Discussion. The relevance of the results consists in this finding: metabolic and cardiovascular problems coexist despite renal denervation, explaining why renal sympathectomy in humans with metabolic syndrome does not provide a therapeutic solution in the long term. It is likely that endocrine players (such as leptin) outdo the autonomic regulation and might contribute severely to the metabolic syndrome physiopathology.

Reviewer #2 (Comments to the Authors (Required)):

The manuscript by Flores-Sandoval and colleagues addresses an important topic with clear translational relevance, namely the autonomic, cardiovascular, metabolic, and renal consequences of chronic exposure to a high-fat diet (HFD). The study provides several noteworthy findings. First, HFD induces a progressive and sustained mild hypertension, affecting systolic, diastolic, and mean blood pressure. Second, renal denervation exerts a modest and transient effect, primarily influencing systolic blood pressure and circulating angiotensin II levels during intermediate stages. Third, with prolonged exposure, the effects of a chronic hypercaloric diet appear to override autonomic modulation, consistent with the progression of metabolic syndrome.

In addition, the data support the concept that dietary fat intake is the predominant driver of adiposity and metabolic disturbances. Renal denervation shows limited and short-lived

metabolic effects, particularly with respect to triglyceride regulation. Overall, the findings reinforce the broader conclusion that autonomic modulation plays a relatively minor role in long-term metabolic outcomes under sustained HFD conditions.

The manuscript is well presented, and the results are interesting and generally well supported. Nevertheless, several concerns should be addressed to strengthen the conclusions:

1. The authors state that renal sympathetic activity was evaluated; however, sympathetic nerve activity was not directly recorded. Therefore, it should be clarified that the study assesses the effects of total renal denervation on the measured outcomes, rather than direct measurements of renal sympathetic nerve activity (RSNA).

REPLY:

We thank the Reviewer for highlighting this inconsistency. We did not perform any electrophysiological investigation because it was beyond the purpose of the present study. On the other hand, renal noradrenaline content has already been used as an index of renal denervation accomplishment. This method has been applied by Mühlbauer et al., [Am J Physiol., 1997 (https://doi.org/10.1152/ajprenal.1997.273.1.F144)] and Rodionova et al., [Am J Physiol Regul Integr Comp Physiol. 2016 (10.1152/ajpregu.00227.2014)], already cited in the manuscript. To avoid further confusion, the word "activity" has been replaced with more appropriate terms, when possible.

2. The renal nerve contains both efferent and afferent fibers. The manuscript would benefit from a discussion of the relative contribution of afferent versus efferent signaling to the observed effects. In this context, the potential role of TRPV1-expressing afferent fibers in mediating cardiovascular and renal responses should be addressed.

REPLY:

- We apologize for the inaccurate description of the surgical procedure, which has been improved. The whole bunch of nerve fibers running along the renal artery has been retired, from the aortorenal ganglion to the renal hilum. In addition, according to Cheng et al. [J Am Soc Nephrol. 2022 (https://doi.org/10.1681/ASN.2021111518)] parasympathetic innervation from the renal plexus also reaches the kidney via the same route, although separated by the sympathetic and sensory fibers. For that, we consider that the sympathetic efferents (which represent the main portion of renal

innervation), the parasympathetic (vagal?) fibers, and visceral polymodal sensory afferents were most likely removed.

- We thank the Reviewer questioning the effect of sympathetic renal innervation on the visceral sensitivity. This point has already indicated a possible contraindication for renal denervation in human patients, since it is unknown whether this procedure also dampens the kidney's ability to detect noxious stimuli and what consequences this may have in the long term.
- Chemical ablation of sensory fibers has been reported to decrease Na^+ sodium excretion in overt hypertension and after salt loading [Wang et al. Am J Physiol Heart Circ Physiol. 2005 (doi:10.1152/ajpheart.00370.2005)]. This is just the opposite of what occurs in denervated animals sacrificed at 8-weeks, which are supposed to lose the renal sensory function. These rats were fed with either the hypercaloric or the normal diet, but with a normal Na^+ content, and had a normal urinary [Na^+]. On the other hand, the decrease in Na^+ sodium excretion has been observed only in intact animals fed with the hypercaloric diet for 8 weeks. This is consistent with the suggestion that, *in vivo*, more than one stimulus must be present simultaneously to activate TRPV1 [Blumberg et al. Curr Top Med Chem. 2011 (doi: 10.2174/156802611796904825)].
- In our experiments, the virtual removal of the afferent unmyelinated fibers does not allow us to speculate further about TPV1-R because there is such several concurring neural and humoral players that any conclusion would be premature.

This limitation and the potential role of TRPV1-mediated sensory signaling have now been discussed in the revised Discussion section

3. Renal denervation was assessed by measuring noradrenaline content in renal tissue. While this is an accepted approach, complementary evaluation of tyrosine hydroxylase expression would strengthen the validation of effective denervation.

REPLY:

As already mentioned, we measured renal noradrenaline content to corroborate the effectiveness of the surgical procedure on the basis of what is indicated in the literature. We agree with the Reviewer: other methodological approaches could strengthen the bases of the present work. However, the results of noradrenalin quantification were consistent in the

whole set of denervated rats, thus we went for using our resources for the following experiments.

4. Blood pressure was measured using an indirect tail-cuff method, which has known limitations in accuracy and sensitivity in rodents. The authors should discuss whether the observed effects would be expected to persist if blood pressure were measured using direct intra-arterial recordings.

REPLY:

We concur with the Reviewer: intra-arterial and even telemetry recording might provide more accurate results with respect to the non-invasive tail-cuff measurement. However, in the present study blood pressure was measured using the non-invasive tail-cuff method, which is widely used for longitudinal studies in rats and allows repeated measurements over extended experimental periods without additional surgical procedures. Because the denervated rats had already undergone an abdominal surgery, and any other invasive intervention could only add unnecessary risks. Under these conditions, the tail-cuff method allowed consistent monitoring of systolic, diastolic, and mean blood pressure throughout the experimental protocol. We have now clarified this point and acknowledged this methodological limitation in the revised Materials and methods section.

5. The manuscript reports a lower water intake in the standard diet (SD) group. A mechanistic explanation for this finding should be provided or discussed.

REPLY:

The decreased water intake in HFD-animals has been discussed together with its effect on K^+ and Na^+ excretion. Briefly, a similar behavior has been observed in human obese subjects and may be explained by the interplay of several factors, including the change in body volume and the osmolar sensitivity, as well as by the upregulation of the renin-angiotensin system and all the consequences on arterial caliber and hormone release.

6. Renal denervation reduced circulating angiotensin II levels in HFD-fed animals at 8 weeks, but not at 12 weeks. How do the authors explain this temporal difference? Could renal nerve reinnervation or compensatory activation of other components of the renin-angiotensin system account for this effect?

REPLY:

The temporal difference in A-II levels in HFD-rats between the group sacrificed at 8 weeks and 12 weeks has been examined in the paragraph that discusses the renal denervation inefficiency in preventing the hormonal complication of the unbalanced diet. Briefly, besides the canonical renin-angiotensin system (RAS), it is also necessary to consider the upregulation triggered by the HFD in the local RAS distributed in many body organs and tissues, such as the adipose tissue.

7. Given that high-fat intake itself can directly promote renal injury, the authors should clarify how they distinguish the contributions of renal nerves and/or angiotensin II from the direct effects of HFD on blood pressure, metabolic parameters, renal function, and intracellular signaling pathways (Akt, PI3K, and ERK1/2).

We thank the reviewer for this observation. To investigate a possible molecular process linking the metabolic changes caused by a high-fat diet with intracellular responses in the kidney, the evaluation of intracellular signaling pathways (PI3K, Akt, and ERK1/2) was indeed included in this study. Angiotensin II, leptin, insulin, and other metabolic mediators that are modified in obesity and metabolic syndrome converge in these intracellular pathways, as discussed in the manuscript. Specifically, leptin signaling has been shown to stimulate PI3K-Akt and ERK cascades, as well as mesangial cell hypertrophy and fibrogenic processes in the kidney. As an indication of intracellular signaling that may be influenced by both metabolic status and renal sympathetic activity, the phosphorylation status of PI3K, Akt, and ERK was examined.

However, the results show a rather complex pattern in which various signaling pathways are influenced by both sympathetic influence and HFD. For example, denervated animals fed a normal diet show a significant increase in the PI3K pathway, while denervated animals fed an HFD show a decrease. This suggests that renal sympathetic input can alter PI3K signaling depending on metabolic status. Similarly, nutrition and renal sympathetic activity appear to influence ERK phosphorylation. Therefore, these results show that intracellular signaling in the kidney exhibits a combined effect of metabolic and neurological signaling. However, at this point, the data do not allow us to thoroughly analyze the relative impact of each component.

June 16, 2026

RE: Life Science Alliance Manuscript #LSA-2025-03568-TR

Dr. Nadia Saderi
Autonomous University of San Luis Potosí
Laboratorio de Neuroanatomía Funcional, Facultad de Ciencias
Av. Parque Chapultepec 1570, Privadas del Pedregal
San Luis Potosí, San Luis Potosí 78295
Mexico

Dear Dr. Saderi,

Thank you for submitting your revised manuscript entitled "The onset of hypertension in metabolic syndrome is independent of renal sympathetic innervation". As you will see, the reviewers are now satisfied with no further requests. We would be happy to publish your paper in Life Science Alliance pending final revisions necessary to meet our formatting guidelines.

MANUSCRIPT ORGANIZATION AND FORMATTING:

To avoid unnecessary delays in the acceptance and publication of your paper, please read the following information carefully. Full guidelines are available on our Instructions for Authors page, https://www.life-science-alliance.org/authors

Submission of a paper that does not conform to Life Science Alliance guidelines will delay the acceptance of your manuscript.

-Please add ORCID ID for corresponding author - you should have received instructions on how to do so.
-Abstract should be a single paragraph not exceeding 175 words and must match between the system and the manuscript file.
-Please add a Summary Blurb/Alternate Abstract and a Category for your manuscript in our system.
-Please add the X and Bluesky handles of your host institute/organization, as well as your own, and/or one of the authors, in our system.
-On the title page of the manuscript, please provide the full name of each author formatted as follows: First name, middle initial, Last name.
-Please rename the "Competing interest" section to "Conflict of interest."
-Please remove the section "Supplementary material" from your manuscript text.

We encourage our authors to provide original source data, particularly uncropped/-processed electrophoretic blots and spreadsheets for the main figures of the manuscript. If you would like to add source data, we would welcome one PDF/Excel-file per figure for this information. These files will be linked as supplementary "Source Data" files.

It is Life Science Alliance policy that if requested, original data images must be made available to the editors. Failure to provide original images upon request will result in unavoidable delays in publication. Please ensure that you have access to all original data images prior to final submission.

If you are planning a press release on your work, please inform us immediately to allow informing our production team and scheduling a release date.

We welcome submissions of potential cover images for the issue of LSA in which your work would appear. If you have high quality images associated with this work, please feel free to email these, with a caption, to the journal office.

LSA encourages authors to provide a 30-60 second video where the study is briefly explained. These videos will be appear embedded with the manuscript online at Life Science Alliance, and on social media to promote the published paper and authors (for examples, see https://docs.google.com/document/d/1-UWCfbE4pGcDdcgzcmiuJl2XMBJnxKYeqRvLLrLSo8s/edit?usp=sharing). Corresponding or first-authors are welcome to submit the video. Please submit only one video per manuscript. The video can be emailed to contact@life-science-alliance.org

FINAL FILES:

To upload the final version of your manuscript, please log in to your account: https://lsa.msubmit.net/cgi-bin/main.plex
You will be guided to complete the submission of your revised manuscript and to fill in all necessary information. Please get in touch in case you do not know or remember your login name.

The following items are required for acceptance.

-- An editable version of the final text (.DOC or .DOCX) is needed for copyediting (no PDFs).

-- High-resolution figure, supplementary figure and video files uploaded as individual files: See our detailed guidelines for preparing your production-ready images, https://www.life-science-alliance.org/authors

-- Summary blurb (enter in submission system): A short text summarizing in a single sentence the study (max. 200 characters including spaces). This text is used in conjunction with the titles of papers, hence should be informative and complementary to the title. It should describe the context and significance of the findings for a general readership; it should be written in the present tense and refer to the work in the third person. Author names should not be mentioned.

The license to publish form must be signed before your manuscript can be sent to production. A link to the license to publish form will be available to the corresponding author only. Please take a moment to check your funder requirements.

Reviews, decision letters, and point-by-point responses associated with peer-review at Life Science Alliance will be published, alongside the manuscript. If you do want to opt out of having the reviewer reports and your point-by-point responses displayed, please let us know immediately.

Thank you for your attention to these final processing requirements. Please revise and format the manuscript and upload materials as soon as you are able.

Thank you for this interesting contribution to the literature. We look forward to publishing your paper in Life Science Alliance.

Sincerely,

Tim Fessenden
Scientific Editor
Life Science Alliance

Reviewer #1 (Comments to the Authors (Required)):

The authors have addressed all of my comments

Reviewer #2 (Comments to the Authors (Required)):

The authors have revised the manuscript in accordance with my comments, and I have no further comments.

June 29, 2026

RE: Life Science Alliance Manuscript #LSA-2025-03568-TRR

Dr. Nadia Saderi
Autonomous University of San Luis Potosí
Laboratorio de Neuroanatomía Funcional, Facultad de Ciencias
Av. Parque Chapultepec 1570, Privadas del Pedregal
San Luis Potosí, San Luis Potosí 78295
Mexico

Dear Dr. Saderi,

Thank you for submitting your Research Article entitled "The onset of hypertension in metabolic syndrome is independent of renal sympathetic innervation". It is a pleasure to let you know that your manuscript is now accepted for publication in Life Science Alliance. Congratulations on this interesting work.

The final published version of your manuscript will be deposited by us to PubMed Central upon publication.

Your manuscript will now progress through copyediting and proofing. It is journal policy that authors provide original data upon request.

Your article will publish open access upon publication under a CC-BY license.

Reviews, decision letters, and point-by-point responses associated with peer-review at Life Science Alliance will be published, alongside the manuscript. If you do want to opt out of having the reviewer reports and your point-by-point responses displayed, please let us know immediately.

IMPORTANT: If you will be unreachable at any time, please provide us with the email address of an alternate author. Failure to respond to routine queries may lead to unavoidable delays in publication.

Scheduling details will be available from our production department. You will receive proofs shortly before the publication date. Only essential corrections can be made at the proof stage so if there are any minor final changes you wish to make to the manuscript, please let the journal office know now.

DISTRIBUTION OF MATERIALS:
Authors are required to distribute freely any materials used in experiments published in Life Science Alliance. Authors are encouraged to deposit materials used in their studies to the appropriate repositories for distribution to researchers.

You can contact the journal office with any questions, contact@life-science-alliance.org

Again, congratulations on a very nice paper. I hope you found the review process to be constructive and are pleased with how the manuscript was handled editorially. We look forward to future exciting submissions from your lab.

Sincerely,

Tim Fessenden
Scientific Editor
Life Science Alliance
